# Incidence, Clinico-demographic Profiles and Survival Rates of Colorectal Cancer in Northern Malaysia: Comparing Patients Above and Below 50 Years of Age

**DOI:** 10.31557/APJCP.2020.21.4.1057

**Published:** 2020-04

**Authors:** Nik Razima Wan Ibrahim, Huan-Keat Chan, Shahrul Aiman Soelar, Ahmad Najib Azmi, Rosaida Mohd Said, Muhammad Radzi Abu Hassan

**Affiliations:** 1 *Gastroenterology Unit, Department of Medicine, Ampang Hospital, Ampang, Selangor, *; 2 *Clinical Research Center, Sultanah Bahiyah Hospital, Alor Setar, *; 3 *Faculty of Medicine and Health Sciences, Islamic Science University of Malaysia, Kuala Lumpur, *; 4 *Gastroenterology Unit, Department of Medicine, Sultanah Bahiyah Hospital, Alor Setar, Malaysia. *

**Keywords:** Colorectal neoplasms, early detection of cancer, Malaysia, survival rate

## Abstract

**Background:**

While the world witnesses an increasing trend of young-onset colorectal cancer (CRC), the information regarding the impact of age on CRC is limited in Malaysia. This study aimed to compare the incidence, clinic-demographic profiles and survival rates of CRC between patients above and under 50 years of age in northern Malaysia.

**Methods:**

This was a registry-based, cross-sectional study. All the CRC cases reported by 18 hospitals to the National Cancer Patient Registry - Colorectal Cancer (NCPR-CC) between January 2007 and December 2017 were included in the analysis. The patients were categorized by age into the above-50 and under-50 groups. The changes in the age-standardized incidence and mortality rates of both the age groups were determined using the time-series analysis, and the impact of age on the mortality risk was assessed using the Cox regression analysis.

**Results:**

Of the 6,172 CRC patients enrolled in the NCPR-CC, 893 (14.5%) were in the under-50 group. As compared with their older counterparts, the patients in the under-50 group were more likely to be female, be of Malay ethnicity, be non-smokers, have a family history of CRC, and present late for treatment. The age-standardized incidence and mortality rates of CRC in the under-50 group remained stable over the years, while a decreasing trend was clearly seen in the mortality rates of CRC in the above-50 group (p=0.003). Nevertheless, the two age groups also did not differ in the mortality risk (adjusted hazards ratio: 1.10; 95% CI: 0.90, 1.36).

**Conclusion:**

Young-onset CRC constituted a considerable proportion of CRC cases in Malaysia. However, in contrast with the findings of most studies, it demonstrated neither an uptrend in age-standardized incidence rates nor a higher mortality risk. Our findings suggest the need to upscale and lower the recommended age for CRC screening in Malaysia.

## Introduction

Colorectal cancer (CRC) is the third most common cancer in men and the second in women worldwide. In 2018, the World Health Organization reported nearly 1.85 million new CRC cases and 0.88 million CRC-related deaths (Wong et al., 2019). Malaysia, together with China, Japan, Korea and Singapore, has recorded the highest 5-year prevalence of CRC (≥46.5 cases in 100,000) in Asia (Haggar and Boushey, 2009).

It is well known that the prognosis of a CRC patient is strongly related to the stage at which the cancer is diagnosed. The 5-year survival rate of CRC is higher than 80% for stage 1 and 2, between 30 and 60% for stage 3, and lower than 10 % for stage 4 (Rashid et al., 2009). Although the overall 5-year survival rate of CRC in Malaysia had considerably increased from 40% in the late 1990’s to 53% in the first 5 years after the millennium (Ghazali et al., 2010; Kong et al., 2010), most CRC patients are still diagnosed only at late stages (Veettil et al., 2017).

The incidence of CRC has also been shown to disproportionately increase with age, with 90% of the cases occurring in individuals aged 50 years and above (Feletto et al., 2019). Therefore, CRC screening, mainly by using colonoscopy or fecal occult blood testing (FOBT), is highly recommended for this age group globally. Large-scale screening programs and media campaigns have successfully driven the growth in the uptake of CRC screening, notably in western countries. In the US alone, the uptake of annual CRC screening in individuals aged between 50 to 74 years had increased from 51.6% in 2008 to 61.3% in 2015 (de Moor et al., 2018). Early detection and removal of tumors or adenomatous polyps, along with the improved health consciousness and advances in cancer treatment, had resulted in a 30% decline in both the global incidence and mortality of CRC between 2003 and 2012 (Kopetz et al., 2009; Cone et al., 2011; Edwards et al., 2010; Siegel et al., 2012; Siegel et al., 2014; Siegel et al., 2017).

Ironically, since the early 1990s, the US has also been witnessing an annual increase of nearly 2% in the incidence of CRC among individuals under 50 years of age (Myers et al., 2019). Delayed presentation for treatment is common in young CRC patients (Fu et al., 2014). Yet, regular CRC screening in young individuals remains controversial and is often deemed to be less cost-effective. Despite the same concern raised about the early-onset CRC in Malaysia (Veettil et al., 2017), the information regarding this issue is still limited. As an initial step to fill this gap, this study was designed to compare the incidence, clinic-demographic profiles and survival rates of CRC between patients above and under 50 years of age in northern Malaysia. 

## Materials and Methods

This registry-based, cross-sectional study focused on three states in northern Malaysia (Perlis, Kedah and Penang), as all the 18 public and private hospitals in this region have been reporting all the CRC cases encountered by them to the National Cancer Patient Registry - Colorectal Cancer (NCPR–CC) since its inception back in 2007. All the CRC patients enrolled in the NCPR-CC between January 2007 and December 2017 were included in this the analysis. The patient information gathered from the NCPR-CC included age, gender, ethnicity, smoking status, diabetes mellitus (DM) status, family history of CRC, symptoms at presentation, treatment, cancer stage at diagnosis, and survival status up until December 2017. 

The patients were subsequently dichotomized by age into the above-50 (≥50 years of age) and under-50 (<50 years of age) groups. For each age group, based on the population data provided by the Department of Statistics (2010), the crude and age-standardized incidence and mortality rates of CRC from 2007 to 2017 were calculated and presented as the number of cases in 100,000 (Ahmad et al., 2010). Their changes over the years were subsequently assessed using the time series analysis. Furthermore, the demographic and clinical profiles of the two age groups were summarized as frequencies and percentages, and were compared using the Pearson’s chi-square tests of independence. Apart from that, the 3- and 5-year survival rates of both the age groups were expressed as percentages and 95% confidence intervals (CIs). The impact of age on the mortality risk was also assessed using the Cox regression analysis, with the results summarized as hazards ratios (HRs) and 95% CIs. The data analysis was performed using the R-3.5.1 for Windows. All the statistical tests were two-sided, with the significant level fixed at 5%. 

## Results

Over the 11-year period studied, a total of 6,172 CRC cases were reported to the NCPR-CC. Eight hundred ninety-three (14.5%) of the patients were under 50 years of age, and nearly 70% of them were in the age range of 40 to 49 years. The overall age-standardized incidence rates of CRC were 169.86 and 25.23 cases in 100,000 for the above-50 and under-50 groups, respectively. The above-50 group also recorded a higher overall age-standardized mortality rate as compared with the under-50 group (95.57 vs. 12.17 cases in 100,000). However, only the above-50 group demonstrated a decreasing trend in the mortality rates over years (p=0.003) (Table 1). 

Most of the CRC patients were male (54.1%), of Chinese ethnicity (56.8%) and non-smokers (29.3%). Only 3.0% of them had a family history of CRC. However, as compared with the above-50 group, the under-50 group was more likely to be female (49.6% vs. 45.2%; p=0.015), be of Malay ethnicity (50.7% vs. 33.4%; p<0.001), be non-smokers (33.0% vs. 28.7%; p<0.001), and have a family history of CRC (5.4% vs. 2.6%; p<0.001). The most common symptoms at presentation included the alteration of bowel habit (31.4%), abdominal or anal pain (25.6%), and the presence of blood in the stool or rectal bleeding (25.3%). Abdominal or anal pain (29.8% vs. 24.9%; p=0.002) and intestinal obstruction (7.4% vs. 5.5%; p=0.025) were more commonly seen in the under-50 group. DM, on the other hand, was more prevalent in the above-50 group (18.5% vs. 7.3%; p<0.001) (Table 2). 

In both the age groups, more than 60% of the patients first presented with left-sided CRC. Nevertheless, the under-50 group was more likely to be diagnosed at advanced stages (III or IV) (45.6% vs. 38.3%; p=0.002) of CRC, as well as to receive chemotherapy and biological therapy (49.5% vs. 31.8%; p<0.001) and radiotherapy (17.2% vs. 13.3%; p=0.001) (Table 2). Yet, the two age groups did not considerably differ in both the 3- and 5-year survival rates. Additionally, it is found that the age of patients, dichotomized by using 50 years as the cut-off, was not a significant predictor of mortality (adjusted hazards ratio: 1.10; 95% CI: 0.90, 1.36; p=0.359) (Table 3).

**Table 1:** Incidence and Mortality Rates (Number of Cases in 100,000) by Age of Colorectal Cancer Patients, 2007-2017

Variables	Overall, n=6,172	Year											Time-series analysis		
		2007 n=426	2008 n=575	2009 n=590	2010 n=601	2011 n=537	2012 n=536	2013 n=548	2014 n=553	2015 n=618	2016 n=634	2017 n=554	β	*P*-value	Trend
	
Incidence (in 100,000)															
Under-50 group															
CR	24.96	1.84	2.54	2.26	2.71	1.84	2.07	2.29	2.54	2.38	2.21	2.26	0.011	0.700	Stable
ASR	25.23	1.85	2.57	2.26	2.72	1.87	2.12	2.30	2.58	2.41	2.26	2.29	0.014	0.618	Stable
Above-50 group															
CR	147.54	10.06	13.53	14.23	14.09	13.16	12.91	13.02	12.91	14.90	15.51	13.22	0.211	0.118	Stable
ASR	169.86	11.61	15.64	16.55	16.19	15.18	14.92	15.02	14.86	16.86	17.76	15.28	0.227	0.137	Stable
Mortality (in 100,000)															
Under-50 group															
CR	12.02	0.87	1.31	1.20	1.54	1.03	1.17	1.23	1.51	1.09	0.75	0.31	-0.048	0.168	Stable
ASR	12.17	0.87	1.34	1.20	1.53	1.05	1.20	1.24	1.54	1.11	0.78	0.30	-0.048	0.168	Stable
Above-50 group															
CR	81.64	7.32	9.70	10.28	9.31	8.44	8.13	7.24	6.74	6.99	5.23	2.26	-0.047	0.182	Stable
ASR	95.57	8.57	11.39	12.14	10.85	9.92	9.55	8.46	7.81	8.07	6.13	2.68	-0.540	0.003	Decreasing

CR, crude rate; ASR, age-standardized rate.

**Table 2:** Demographic and Clinical Characteristics of the under-50 and above-50 Groups, 2007-2017

Variables	All patients,		Age groups				*P*-value ^b^
	n=6, 172 ^a^		<50 years n=893		≥50 years n=5,279		
	N	(%)	n	(%)	n	(%)	
Gender							0.015
Male	3,342	(54.1)	450	(50.4)	2,892	(54.8)	
Female	2,830	(45.9)	443	(49.6)	2,387	(45.2)	
Ethnicity							<0.001
Malay	2,216	(35.9)	453	(50.7)	1,763	(33.4)	
Chinese	3,503	(56.8)	348	(39.0)	3,155	(59.8)	
Indian	382	(6.2)	74	(8.3)	308	(5.8)	
Others	71	(1.2)	18	(2.0)	53	(1.0)	
Smoking status							<0.001
Never	1,811	(29.3)	295	(33.0)	1,516	(28.7)	
Quitting ≥30 days	330	(5.3)	67	(7.5)	263	(5.0)	
Active	500	(8.1)	47	(5.3)	453	(8.6)	
Family history of colorectal cancer							<0.001
No	3,075	(49.8)	431	(48.3)	2,644	(50.1)	
Yes	186	(3.0)	48	(5.4)	138	(2.6)	
Symptoms at presentation							
Alteration of bowel habit	1,937	(31.4)	278	(31.1)	1,659	(31.4)	0.860
Abdominal or anal pain	1,583	(25.6)	266	(29.8)	1,317	(24.9)	0.002
Blood in the stool or rectal bleeding	1,564	(25.3)	216	(24.2)	1,348	(25.5)	0.392
Weight loss	1,124	(18.2)	163	(18.3)	961	(18.2)	0.972
Anemia	430	(7.0)	57	(6.4)	373	(7.1)	0.459
Appetite loss	427	(6.9)	53	(5.9)	374	(7.1)	0.211
Intestinal obstruction	356	(5.8)	66	(7.4)	290	(5.5)	0.025
Treatment received							
Surgery	4,523	(73.3)	676	(75.7)	3,847	(72.9)	0.078
Chemotherapy and biological Therapy	2,120	(34.3)	442	(49.5)	1,678	(31.8)	<0.001
Radiotherapy	854	(13.8)	154	(17.2)	700	(13.3)	0.001
Supportive or palliative care	270	(4.4)	30	(3.4)	240	(4.5)	0.109
Diabetes mellitus status							<0.001
No	2,883	(46.7)	503	(56.3)	2,380	(45.1)	
Yes	1,042	(16.9)	65	(7.3)	977	(18.5)	
Primary cancer site							0.274
Left side	4,047	(65.6)	562	(62.9)	3,485	(66.0)	
Right side	554	(9.0)	72	(8.1)	482	(9.1)	
Both sides	104	(1.7)	9	(1.0)	95	(1.8)	
Cancer stage at diagnosis							0.002
I	416	(6.7)	52	(5.8)	364	(6.9)	
II	1,081	(17.5)	135	(15.1)	946	(17.9)	
III	1,311	(21.2)	206	(23.1)	1,105	(20.9)	
IV	1,121	(18.2)	201	(22.5)	920	(17.4)	

^a^, Incomplete information is reported for smoking status (n=3,531; 57.2%); family history of colorectal cancer (n=2,911; 47.2%), diabetes mellitus status (n=2,247; 36.4%), primary cancer site (n=1,467; 23.8%) and cancer stage at diagnosis (2,243, 36.3%); ^b^, Pearson's chi-square test of independence.

**Table 3:** Survival Rates of the under-50 and above-50 Groups and Results of Survival Analysis, 2007-2017

Variables	Survival Rate, %		Simple Cox Regression		Multiple Cox Regression ^a^	
	3 years (95% CI)	5 years (95% CI)	Crude HR (95% CI)	p-value	Adjusted HR (95% CI)	p-value
Age groups						
<50 years	60.04 (54.0,66.8)	48.41 (42.0,55.9)	1.00 (ref.)		1.00 (ref.)	
≥50 years	59.52 (56.9,62.3)	50.67 (47.8,53.7)	1.03 (0.84,1.25)	0.801	1.10 (0.90,1.36)	0.359

CI, confidence interval; HR; hazards ratio; ^a^, Adjusted for gender, ethnicity, smoking status, family history of colorectal cancer, symptoms at presentation, treatment received, diabetes mellitus status, primary cancer site and cancer stage at diagnosis. A total of 1,563 patients with complete documentation for all the variables listed were included in the model.

## Discussion

Although the incidence of CRC remarkably increases after the fifth decade of life, the proportion of patients under 50 years of age has increased to more than 10% worldwide (Fu et al., 2014). In agreement with the previous findings, our study shows that 14.5% of the CRC patients in northern Malaysia were in the young age group.

Yet, it is noteworthy that the actual number of patients aged below 50 years could be underestimated in our study. Instead of an uptrend in the incidence of early-onset CRC as shown in most countries (Myers et al., 2019), our study reports a stable trend between 2007 and 2017. This could be attributed to the under-diagnosis of CRC in the young population. In Malaysia, CRC screening has only been highly recommended for older patients, and young individuals are generally regarded as the low-risk group. This could also explain why most young CRC patients only sought treatment at advanced stages of the disease in Malaysia.

While it is widely believed that early-onset CRC is strongly related to genetic factors, our study did find that the under-50 group was more likely to have a family history of CRC. Nonetheless, the proportion of young patients with a family history of CRC shown in our study (5.4%) was much lower than that reported by a recent review (20%) (Mauri et al., 2019). Additionally, it is noted that the young patients had a higher tendency to present late for treatment. Overall, our findings implies under-screening for CRC in the at-risk young population in the country. 

Interestingly, early-onset CRC, consistent with the findings of a local study back in 1998, was found to be more prevalent in the Malay ethnic group, even though the Chinese ethnic group constitutes the largest proportion of CRC cases in Malaysia (Veettil et al., 2017). The ethnic variations in the incidence of early-onset CRC are likely due to the differences in health consciousness and the awareness of the disease. However, more studies are required to verify and explain such findings.

Unlike most studies (Siegel et al., 2019), our study only showed a declining trend in the mortality but not in the incidence rates of CRC in the above-50 group. Moreover, in contrast to the previous findings (Fu et al., 2014), the older patients were not shown to have a lower mortality risk as compared with their younger counterparts. It is clear that the public awareness of CRC is still limited regardless of age in Malaysia, and the uptake of CRC screening in the targeted age group is also yet to be optimized (Hilmi et al., 2010; Al-Naggar and Bobryshev, 2013).

While the Clinical Practice Guidelines of Colorectal Carcinoma recently launched by the Malaysia Health Technology Assessment Section (2017) recommends CRC screening in individuals above 50 years of age, it is noteworthy that approximately 70% of the young patients in our study were in the age range of 40 to 49 years. A similar observation was reported by the US and Taiwan (Chen et al., 2016; Mannucci et al., 2019). All these findings are suggestive of the need for adopting the proposal of the American Cancer Society to further lower the recommended age for CRC screening in Malaysia (Wolf et al., 2018). This can potentially help detect cancer at earlier stages in more patients and, in turn, save more lives. 

As our study is only limited to only three states, a nationwide study is required to comprehensively explore the epidemiology and risk factors of early-onset CRC in the country. Additionally, the analysis was limited to the information reported to the NPPR-CC. Due to the incomplete documentation, only 1,563 patients were included in the Cox regression analysis. While the extension of CRC screening to younger patients is suggested based on our findings, further studies on its cost-effectiveness are also warranted. 

In conclusion, as in many countries, the under-50 group was found to compose a considerable proportion of CRC patients in northern Malaysia. Nevertheless, the age-standardized incidence and mortality rates of CRC in this group was not shown to increase with time. Although the above-50 group showed a decreasing trend in the age-standardized mortality rates over the years as expected, it did not have a lower mortality risk as compared with the under-50 group. Our findings are suggestive of the need to scale up and, if feasible, to lower the recommended age for CRC screening in Malaysia. 
